# Infinium Monkeys: Infinium 450K Array for the Cynomolgus macaque (*Macaca fascicularis*)

**DOI:** 10.1534/g3.114.010967

**Published:** 2014-05-08

**Authors:** Mei-Lyn Ong, Peck Yean Tan, Julia L MacIsaac, Sarah M Mah, Jan Paul Buschdorf, Clara Y Cheong, Walter Stunkel, Louiza Chan, Peter D. Gluckman, Keefe Chng, Michael S. Kobor, Michael J Meaney, Joanna D Holbrook

**Affiliations:** * Singapore Institute of Clinical Sciences (SICS), A*STAR, Brenner Centre for Molecular Medicine, Singapore 117609; † Centre for Molecular Medicine and Therapeutics, Child and Family Research Institute, University of British Columbia, Canada; ‡ Ludmer Centre for Neuroinformatics and Mental Health, Douglas Institute, McGill University, Montreal, Canada

**Keywords:** epigenetics, DNA methylation, nonhuman primates, disease model, cynomolgus macaque, *Macaca fascularis*, Illumina Infinium Human Methylation450 BeadChip Array

## Abstract

The Infinium Human Methylation450 BeadChip Array (Infinium 450K) is a robust and cost-efficient survey of genome-wide DNA methylation patterns. *Macaca fascicularis* (Cynomolgus macaque) is an important disease model; however, its genome sequence is only recently published, and few tools exist to interrogate the molecular state of Cynomolgus macaque tissues. Although the Infinium 450K is a hybridization array designed to the human genome, the relative conservation between the macaque and human genomes makes its use in macaques feasible. Here, we used the Infinium 450K array to assay DNA methylation in 11 macaque muscle biopsies. We showed that probe hybridization efficiency was related to the degree of sequence identity between the human probes and the macaque genome sequence. Approximately 61% of the Human Infinium 450K probes could be reliably mapped to the Cynomolgus macaque genome and contain a CpG site of interest. We also compared the Infinium 450K data to reduced representation bisulfite sequencing data generated on the same samples and found a high level of concordance between the two independent methodologies, which can be further improved by filtering for probe sequence identity and mismatch location. We conclude that the Infinium 450K array can be used to measure the DNA methylome of Cynomolgus macaque tissues using the provided filters. We also provide a pipeline for validation of the array in other species using a simple BLAST-based sequence identify filter.

Biomedical research relies on nonhuman primate models, including the Cynomolgus macaque. Cynomolgus models are used to investigate diseases as diverse as Parkinson (Grabli *et al.* 2013; Potts *et al.* 2014; Riverol *et al.* 2013), influenza (Kobayashi *et al.* 2013; Pham *et al.* 2013), asthma (Cheng *et al.* 2013), diabetes (Kaplan and Wagner 2006), and atherosclerosis (Clarkson *et al.* 2013; Kaplan *et al.* 1996). However, the genome of this important model only was elucidated recently (Higashino *et al.* 2012; Yan *et al.* 2011), and tools for genomics analysis have consequently been lacking.

The rapidly expanding field of epigenomics has the potential to disentangle the influences of inheritance and environment on complex diseases and greatly increase our understanding, as well as provide biomarkers and therapeutics (Michels *et al.* 2013; Mill and Heijmans 2013). Therefore, it is desirable to study the epigenomics of Cynomolgus macaque disease models. Although candidate gene approaches have been reported (Rager *et al.* 2013), genome-wide approaches have been hampered by the lack of suitable tools. Likewise, although high-coverage MeDIP-seq approaches have been reported in the closely related rhesus macaque (Bell *et al.* 2012; Provencal *et al.* 2012), this technique is expensive, generally less quantitative, and has lower resolution.

The Infinium Human Methylation450 BeadChip Array (Infinium 450K) is a widely used tool in epigenetics that provides a robust and cost-efficient way to measure genome-wide DNA methylation patterns. It is a hybridization array designed to probe human genomic sequences (Bibikova *et al.* 2011). The use of a precursor to the Infinium 450K array, the Infinium 27K array, with Cynomolgus macaque tissues has been previously reported (Howard *et al.* 2011). However, the authors did not test its accuracy for this cross-species use. More recently, the Infinium 450K array has been used on great ape species more closely related to humans (chimpanzee, bonobo, gorilla, and orangutans), and probes were filtered for conservation between the human probe sequence and the ape genomic sequence (Hernando-Herraez *et al.* 2013).

We set out to determine the accuracy of the Infinium 450K in macaque tissues by comparing Infinium 450K and reduced representation bisulfite sequenced (RRBS) DNA methylome profiles generated from the same Cynomolgus muscle biopsies. Here we show that with filtering of the probes for good alignment between the human and Cynomolgus macaque genomic sequence, 51% of the array probes achieve a Pearson correlation R of 0.88 and 86% and 69% of the probes agree within 20% and 10% methylation difference respectively. As we can compare sequence identity with actual measurement variation between Infinium and RRBS, we also provide the sequence identity thresholds to achieve accurate Infinium measures and an *in silico* pipeline for converting the Infinium 450K to additional species.

## Materials and Methods

### Samples

Muscle biopsies were performed on eleven adult male cynomolgus macaques under anesthesia with ketamine (7 mg/kg intramuscularly), and muscle samples were taken from the quadriceps region using a 3-mm biopsy punch. A single cruciate stitch using 6/0 PDS was placed at the end of the procedure. Single doses of amoxicillin (7−15 mg/kg) and meloxicam (0.2 mg/kg subcutaneously) were given. All experimental procedures approved by SingHealth Institutional Animal Care and Use Committee.

### Infinium 450K

After extraction of genomic DNA according to standard procedures, 1 μg genomic DNA was bisulfite converted using EZ DNA Methylation™ Gold Kit (Catalogue No.: D5002, Zymo Research). Successful conversion was confirmed via methylation-specific polymerase chain reaction before proceeding with subsequent steps of the Infinium assay protocol. The bisulfite-converted genomic DNA was isothermally amplified at 37° for 22 hr, enzymatically fragmented, purified, and hybridized on an Infinium HumanMethlyation 450 BeadChip (cat. no. WD-314-1002; Illumina Inc.) at 48° for 18 hr, after which the BeadChip was then washed to remove any unhybridized or nonspecific hybridized DNA. Labeled single-base extension was performed on primers hybridized with DNA, and the hybridized DNA was removed. The extended primers were stained with multiple layers of fluorescence, the BeadChip was then coated using a proprietary solution and scanned using the Illumina iScan system. The image data were processed with the Genome Studio Methylation Module software. Further data processing was performed as described previously (Pan *et al.* 2012).

### Reduced representation bisulfite signaling

One microgram of genomic DNA was restriction digested with 60U of *Msp*I (cat. no. R0106S; New England Biolabs) overnight at 37°. The digested product was purified by conventional ethanol precipitation method. End-repair, addition of ‘A’ tail and adapter ligation were performed using the TruSeq DNA LT Sample Prep Kit V2 (cat. no. FC-121-2001; Illumina), according to manufacturer’s instructions with slight modifications. During the adapter-ligation step, 2 μL of Early Access Methylation Adapter oligo (cat. no. ME-100-0010; Illumina) was used instead. The adapter-ligated DNA fragments were subsequently purified with AMPure XP beads (cat. no. A63881; Agencourt), as per manufacturer’s protocol. Purified fragments were then bisulfite treated using the EpiTect Bisulfite Kit (cat. no. 59104; QIAGEN GmbH), according to manufacturer’s instructions. The converted DNA was amplified in a final concentration of 1 U of PfuTurbo Cx Hotstart DNA Polymerase and 250 µM of dNTP mix (cat. no. STR_600412; Strategene), as well as 500 nM each of polymerase chain reaction primers PE 1.0 and 2.0 (Gu *et al.* 2011). The thermocycling condition used was 2 min at 95° for heat activation, and 15 cycles of 30 sec at 95°, 30 sec at 65°, and 1 min at 72°, followed by a 10-min final extension at 72°. The enriched fragments were purified by gel electrophoresis and quantified by Agilent 2100 Bioanalyzer (Agilent Technologies). Sequencing was performed on the Illumina Genome Analyzer IIx platform, as per manufacturer’s instructions. Raw reads were filtered and trimmed (*i.e.*, base quality < 20 were removed, read length < 20 bp were removed and entire read pair is removed if one of the trimmed reads become shorter than 20 bp), and then aligned against the Cynomolgus macaque genome (Yan *et al.* 2011) using RRBSMAP (Xi *et al.* 2012) with default settings (-p 8 -v 0.1 -w 100 -s 12 -D C-CGG). We filtered for CpGs with at least 10x read coverage and estimated their methylation ratios using the python script methratio in RRBSMAP.

### Alignment of Infinium probes to Cynomolgus macaque genome

We used blastn (Altschul *et al.* 1997) to map the 485,512 50 bp infinium probes onto the Cynomolgus macaque genome using an e-value threshold of e^-10^. We used the Burrows-Wheeler Alignment tool (Li *et al.* 2009) to obtain the mismatched base positions and their location with respect to the CpG site being assayed. For each CpG, we checked for corresponding gene name or partial gene name matches between human and Cynomolgus macaque by searching for the closest gene to the CpG site. We further checked for ortholog gene name changes between human and macaque using the gene mapping from ENSEMBL (Hubbard *et al.* 2002).

## Results

### Approximately 61% of human Infinium 450K probes could be reliably mapped to the Cynomolgus macaque genome and contain the CpG site of interest

**Figure 1:**
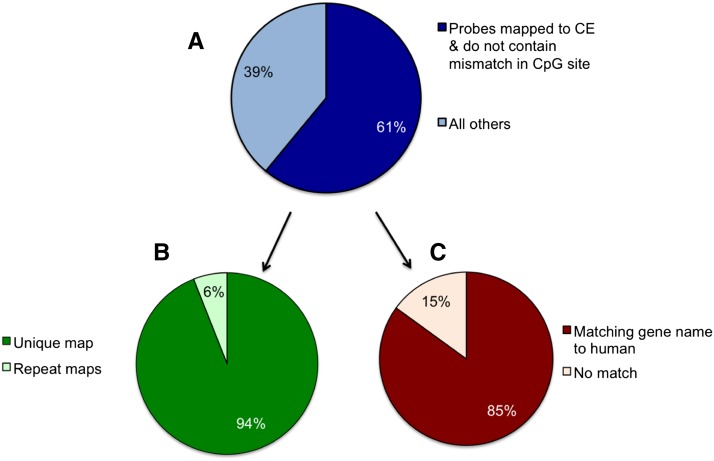
(A) Pie chart (top) showing percent of Infinium 450K probes that can be aligned to the Cynomolgus macaque genome with e-value < e^−10^ and containing the CpG site of interest. (B) Bottom left pie chart shows percent of probes, out of the mapped probes, with unique or repeat matches. (C) Bottom right pie chart shows percent of probes, out of the mapped probes, with gene name matches to human.

We searched the Cynomolgus macaque genome sequence with the 485,512 50-bp Infinium probe sequences using blastn (Altschul *et al.*, 1997) with an e-value threshold of e^−10^. This means that e^−10^ matches would be expected purely by chance and that the candidate match would likely be a *bona fide* match to the probe sequence. We also ran the Burrows-Wheeler Alignment tool (Li *et al.* 2009) to determine the mismatch base positions with respect to the location of the CpG site being assayed. Out of all the human probes, 328,091 probes (68%) could be mapped to the Cynomolgus macaque genome with up to four mismatches and up to two gaps of up to 4 bp. In addition, 30,021 (9.2%) of the mapped probes contained a mismatch in the CpG site itself (including those that do not have the CpG site) and are thus omitted from downstream analyses. We will thereafter refer to this set of mapped probes with the CpG site present as “CM probes” (n = 298,070). A total of 94% of the CM-probes had unique matches, whereas the rest had at least one alignment with e-value < e^−10^ (Figure 1). Thus, in total, approximately 61% of the Human Infinium 450K probes could be reliably mapped to the Cynomolgus macaque genome and contain a CpG site of interest.

### Approximately 85% of CE probes were the Cynomolgus ortholog of the human gene

We then asked whether the gene annotation for the human probes corresponded to the Cynomolgus macaque gene ortholog for the matching macaque sequence. We searched for the exact gene within which the sequence lies or the closest gene to the matching sequence. Of the CM-probes, 229,505 had an associated human gene name from the Illumina Infinium 450K array annotation. Taking into account repeat matches, partial gene name matches and orthologous gene name changes between human and macaque (Hubbard *et al.* 2002), we found that 195,072 (85%) of the CM-probes had the same gene name or the equivalent macaque ortholog of the annotated human gene (Figure 1).

### Cynomolgus tissues run on Infinium 450K arrays passed quality control, and approximately 80% of all probes yielded robust and detectable signal

**Figure 2:**
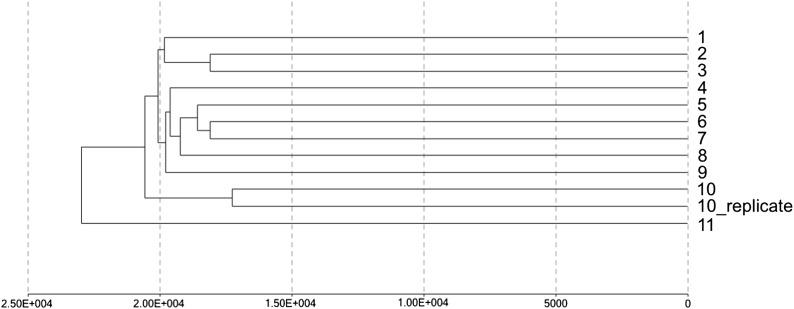
Hierarchical clustering of 12 Infinium 450K data of Cynomolgus macaque muscle tissues with a technical replicate.

We ran the Infinium 450K array on eleven Cynomolgus muscle tissues with one technical replicate. The raw data were analyzed using the Genome Studio Methylation Module software. The Illumina standard controls, including staining, extension, hybridization, target removal, bisulfite conversion, specificity I and II, nonpolymorphic controls all passed quality checks. Similarly, the negative controls, which target bisulfite-converted sequences that do not contain human CpG dinucleotides, and thus measure background intensity, had on average a very low intensity of around 200 AU and 250 AU for the green and red channels respectively (recommended background intensity from Illumina is <300 AU) (Supporting information, Figure S1), suggesting that the negative control probes work well for Cynomolgus macaques. The signal to noise ratio for target probes against negative green and red control probes were, on average, 10.6- and 8.3-fold. The two technical replicates clustered together on the dendogram (Figure 2). The global methylation correlation between the technical replicates was R = 0.97, whereas that between two different muscle samples was R = 0.94 (Figure S2). Notably, on average, 79.1 ± 1.3% of probes across all 12 sample profiles, including the two replicates, had the desired degree of hybridization with a *P*-value detection of less than 0.05 and approximately 73.4 ± 1.6% of probes had a *P*-value detection of less than 0.01. Interestingly, this is for the whole array including probes that do not align well to the Cynomolgus macaque genome, suggesting that some of these non-conserved probes still hybridize efficiently. When the analysis is restricted to CM probes only, on average, 86.8% and 82.4% had a *P*-value of <0.05 and <0.01, respectively. We then ran the Infinium 450K processing pipeline as described by (Pan *et al.* 2012) to obtain the beta values for each CpG on the array.

### The degree of hybridization is positively related to the sequence identity between the human probe and Cynomolgus macaque genome

**Figure 3:**
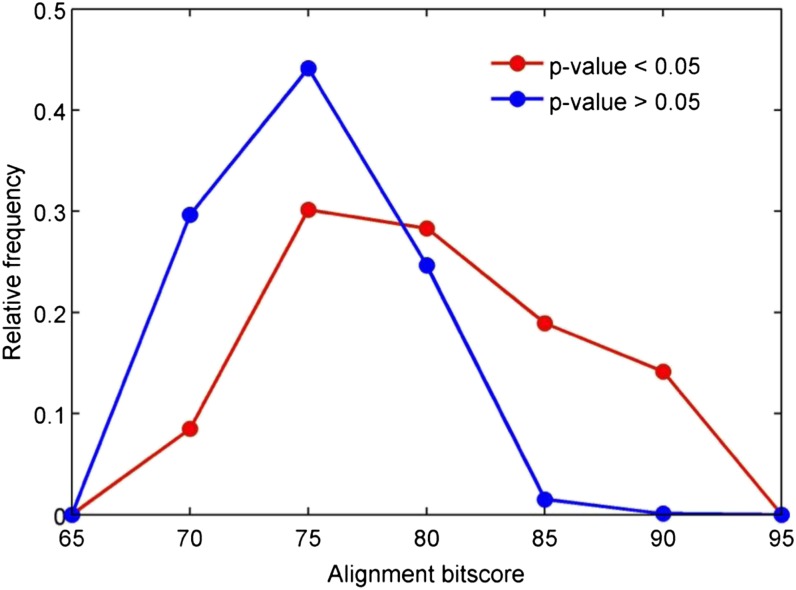
Histograms of alignment bitscore for Infinium probes with detection *P* < 0.05 (red), and > 0.05 (blue).

To assess whether the degree of hybridization of the probes is affected by sequence identity between human and Cynomolgus macaque, we took the CM-probes and computed the correlation between their detection p-values and alignment quality. Detection p-values were positively correlated with alignment e-value scores (Spearman rho = 0.39) and inversely correlated with the percent identity scores and bitscores (Spearman rho = −0.32 and rho = −0.39, respectively). When we excluded probes (n = 21,569) with a mismatch at +/− 1 bp from the CpG site, the correlation between the degree of hybridization and alignment quality improved (Spearman rho = 0.42, −0.36, and −0.42 for e-value, percent identity, and bitscore, respectively). When we grouped probes according to their detection p-values, those with *P* > 0.05 tended to have lower alignment bitscores compared to probes with *P* < 0.05 (Figure 3).

### Concordance of Infinium 450K data and RRBS measurements within Cynomolgus macaque tissues and in relation to probe sequence divergence

**Figure 4:**
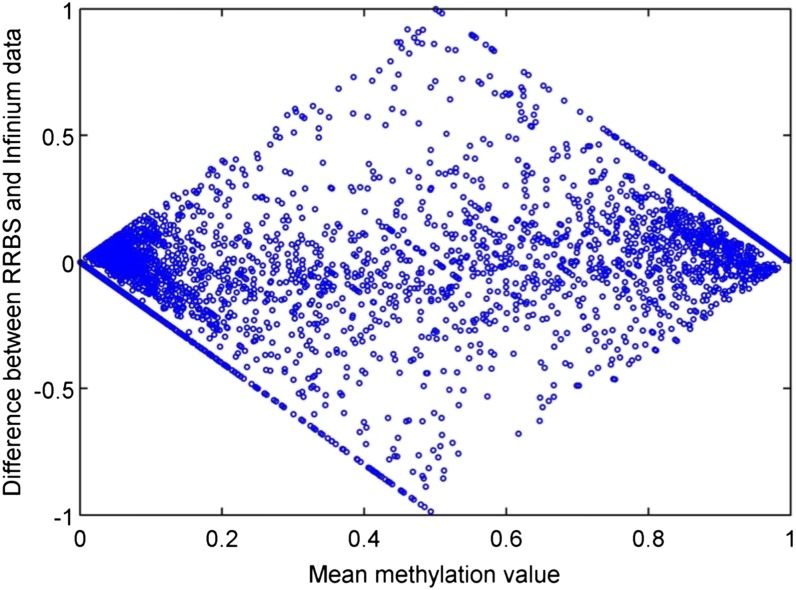
Bland-Altman plot of the difference between reduced representation bisulfite sequenced (RRBS) and Infinium data for one sample *vs.* the average methylation value.

We ran the same 11 Cynomolgus muscle samples using RRBS to determine the degree to which the Infinium 450K data correspond with methylation measurements from an independent sequencing-based technology. We searched each unique sample for CpGs present in both Infinium 450K and RRBS data and found that, on average, there were 4730 such CpGs for each sample. The Bland-Altman plot for one sample shows that the majority of the CpGs lie on *x*-axis (Figure 4). And, consistent with what has been reported previously by Pan *et al.* (2012), CpGs with intermediate methylation levels tend to show the biggest discrepancies between the two technologies.

**Figure 5:**
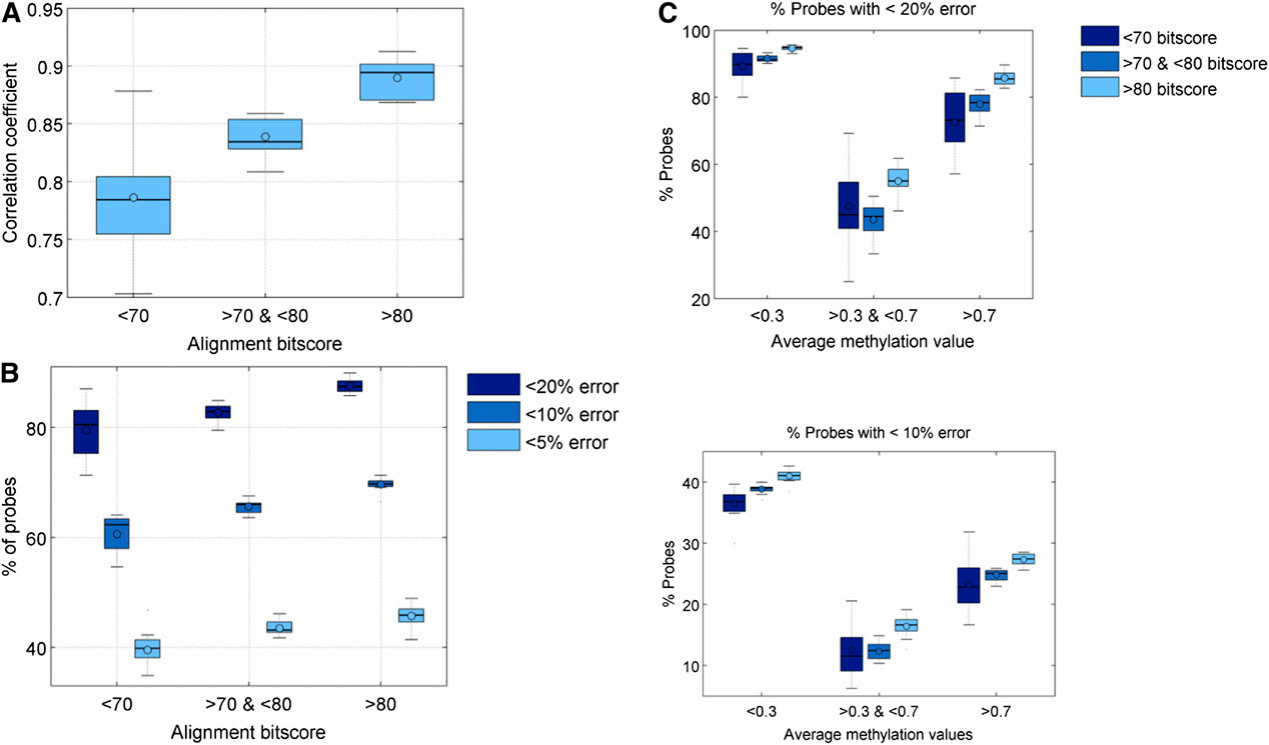
(A) Relationship of correlation between reduced representation bisulfite sequenced (RRBS) and Infinium data with probe sequence divergence (alignment bitscore). (B) Percent of probes with varying percent error between RRBS and Infinium data with probe sequence divergence (alignment bitscore). (C) Percent of probes with <20% error (top) or <10% error (bottom) with varying sequence divergence stratified by their average methylation levels.

To determine the relationship between the degree of agreement of RRBS and Infinium 450K data with sequence divergence, we stratified these shared CM-probe CpGs according to the quality of alignment of the Infinium probes to the Cynomolgus macaque genome. We found that across the 12 paired samples, probes with relatively poorer alignment scores consistently had lower RRBS-Infinium correlations compared with probes with greater alignment scores (Figure 5A). On average, the percent of shared probes with RRBS and Infinium 450K DNA methylation values within 20%, 10%, and 5% of each other were relatively greater for those with greater alignment scores (Figure 5B). The range across individuals is small except at very low alignment bitscores (Table S1). We further stratified CpGs by their average methylation levels and found that the concordance of the majority of unmethylated or methylated CpGs were much better as compared to CpGs with intermediate average methylation levels (Figure 5C).

**Figure 6:**
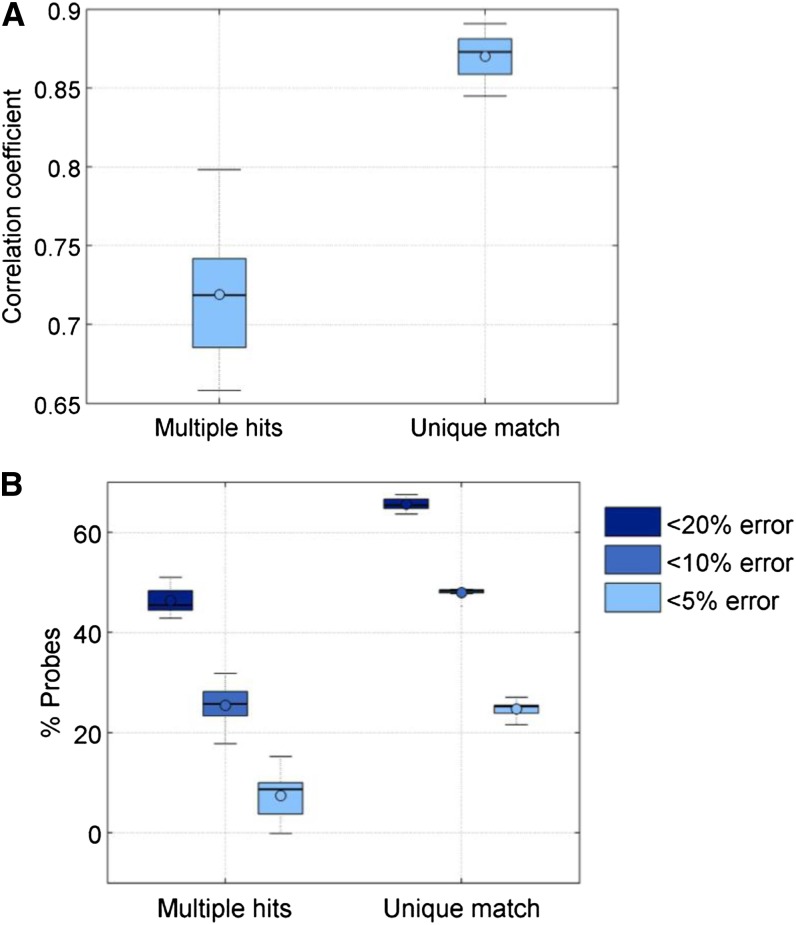
(A) Relationship of correlation between reduced representation bisulfite sequenced (RRBS) and Infinium data for probes with unique alignment or multiple alignments. (B) Percent of probes with varying percent error between RRBS and Infinium data with uniqueness of sequence alignment.

**Figure 7:**
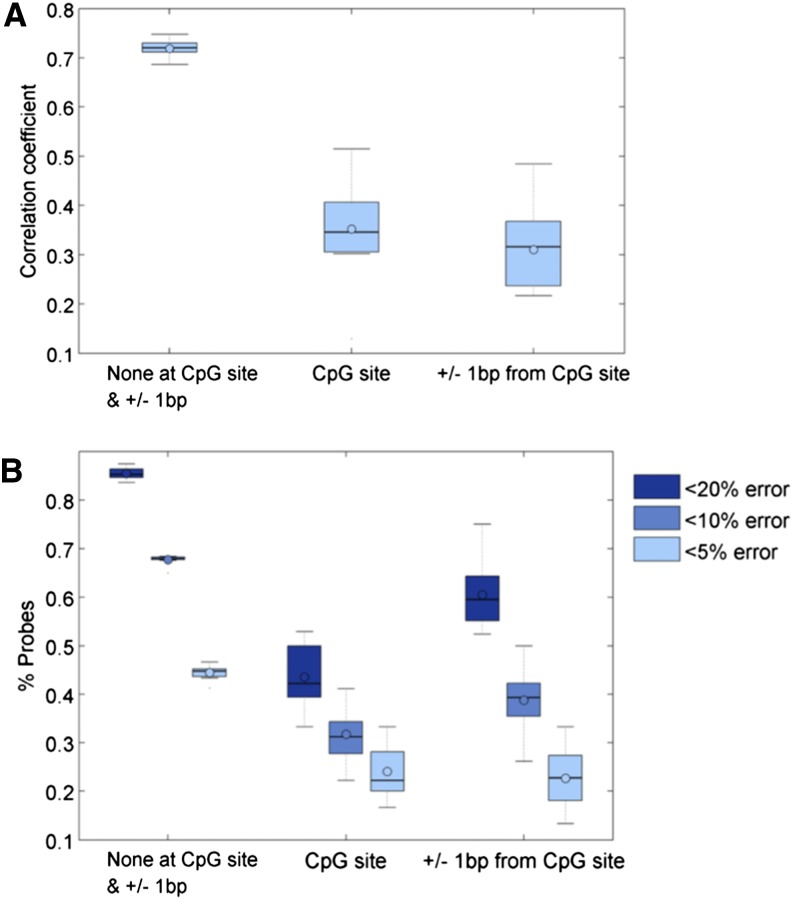
(A) Relationship of correlation between reduced representation bisulfite sequenced (RRBS) and Infinium data for probes with mismatch at specific positions relative to the CpG site of interest. (B) Percent of probes with varying percent error between RRBS and Infinium data with mismatch at specific positions relative to the CpG site of interest.

We also examined whether the concordance between the two technologies is dependent on whether the sequence match is unique. We observe that probes with unique matches tended to have better performance (greater correlation and smaller % error) compared to probes that could be reliably aligned to multiple regions of the Cynomolgus macaque genome (Figure 6). We also examined whether the agreement in methylation values is affected by the position of the mismatch from the CpG site of interest. Probes with no mismatches at both the CpG site and +/− 1 bp had much better correlation and agreement compared with the probes that had either a mismatch at the CpG site itself or close to it. Probes with mismatches at either +/− 1 bp from the CpG tended to perform as badly as probes with mismatches exactly at the CpG site (Figure 7).

We examined whether the concordance between the RRBS and Infinium data differed depending on whether data were from a type I or type II Infinium probe. Type II probes have been shown to have a smaller dynamic range than type I probes (Dedeurwaerder *et al.* 2011), although this effect is corrected in our data processing. We could not detect any difference in the sequence alignment or agreement between RRBS and Infinium data from type I and type II probes (Table S2 and Figure S3). Studies have reported extensive probes that target human polymorphic CpGs in the Infinium 450K array (Chen *et al.* 2013; Price *et al.* 2013). We observed no difference in the RRBS and Infinium 450K agreement between polymorphic and nonpolymorphic probes (Figure S4). In addition to absolute DNA methylation levels, we also analyzed the difference in methylation levels across different individuals measured using the same technology, and compared the concordance of this difference across the two platforms. The differences between inter-individual differences measured on Infinium 450K assays compared to inter-individual differences measured using RRBS were within 20% for 98% of probes, within 10% for 87% of probes and within 5% for 74% of probes (see Figure S5 for an example).

### Probe selection criteria for best concordance and coverage

We observed that the agreement between RRBS and Infinium data can be increased by taking into account sequence divergence of the CM probe, uniqueness of the alignment as well as the position of the mismatch relative to the CpG site being measured. We then assessed the coverage of probes on the array as a function of these three levels of alignment filters and their performance (Table 1). Filtering for uniquely matched probes with no mismatch at 1 bp away from the CpG site and with bitscore >70 (Figure S6), we observed a substantial improvement in the agreement between Infinium and RRBS data, whereas there was a substantial decrease in the number of probes retained at >75 bitscore (Figure S7). When we adjusted the threshold from 60 to 70, the percent of usable probes changed only slightly from 54% to 51% after we applied the three-step filter. As we further increased the bitscore threshold to 75, the performance increased only marginally, but the coverage decreased dramatically to only 41%. Thus, to maximize coverage while maintaining accuracy, we recommend an alignment bitscore threshold of 70.

**Table 1:** Agreement between Infinium and RRBS data in terms of correlation and percent error, and the corresponding of usable Infinium probes after the recommended 3-step filter

	Methylation level												Correlation	Coverage After Sequential Filtering (No. of Probes)			
	<0.3			>0.3 and <0.7			>0.7			All			Pearson’s R	Bit-Score Filter	Uniqueness Filter	Mismatch at ± l-bp Filter	Array Coverage, %
	Error, %																
	<20	<10	<5	<20	<10	<5	<20	<10	<5	<20	<10	<5					
Bitscore <70	89	73	50	47	25	13	73	46	25	80	61	40	0.79				
Bitscore >70	94	81	56	52	30	16	83	53	29	86	69	45	0.88	281673	264,431	245,899	51
Bitscore >80	95	82	57	56	34	17	86	55	31	88	70	46	0.9	154435	146,197	136,039	28

RRBS, reduced representation bisulfite sequenced.

In summary, to obtain reasonably accurate Cynomolgus macaque methylation results from the Human Infinium 450K array, we suggest a three-step probe filter: bitscore >70, uniqueness of sequence match, and no mismatches near to the CpG site. We reason that the use of these procedures should allow for the effective and informative utilization of the Infinium 450K array in species with sequenced genomes. Furthermore, it would be ideal to follow up on the significant results from the array using another method such as pyrosequencing as an additional technical validation. We have provided a comprehensive Cynomolgus macaque annotation file for the Infinium 450K array incorporating all the parameters discussed previously (File S1).

## Discussion

We demonstrated here for the first time that the use of the human Infinium 450K array is feasible in Cynomolgus macaque tissues. Using *in silico* analysis with careful probe filtering, we found that approximately 61% of all human probes could be mapped to the macaque genome and contained an informative CpG, with the vast majority of these probes reporting on orthologous genes. Applying the Infinium 450K array to measure methylation patterns of DNA derived from macaque muscle tissues, we found that a large percentage of all probes passed quality controls and yielded robust DNA methylation signal, which positively correlated with the degree of sequence conservation. Comparing the array-based DNA methylation profiles with those obtained by RRBS provided overall high concordance, suggesting that these technologies can be benchmarked *vs.* one another.

Not surprisingly, we showed that the agreement between RRBS and Infinium 450K data was most dependent on the sequence divergence of the human probes compared with Cynomolgus macaque, the uniqueness of the probe sequence to the Cynomolgus genome, and the position of the mismatch relative to the target CpG. Approximately 250K probes on the array reported less than 20% methylation difference between Infinium and RRBS data. We also observed that the concordance between the two technologies is much better for fully methylated and unmethylated CpGs than the partially methylated CpGs. Finally, we provided a bioinformatics blueprint for the adaptation of Infinium 450K array technology to other species with sequenced genomes.

Our study is a logical extension of previous work using the Infinium 27K array to study DNA methylation in Cynomolgus macaque. The advantages of using the Infinium 450K for such investigations mirror those in human tissues and include the dramatically increased number and broader genomic representation of CpG loci. Others have similarly applied the Infinium 450K array to measure the cytosine methylation profiles of great apes, which are evolutionarily closer to human compared to macaques. From their analysis, approximately 200K (44%) probes were retained for orangutans, which is comparable with the 51% we obtained for Cynomolgus macaque. Great apes are evolutionarily closer to humans than macaques, so the greater percentage of probes retained for macaques might be due to the less-stringent choice of alignment filters in our study. Regardless, these studies collectively open new areas for evolutionary research with broad biological implications. Specifically, using the Infinium 450K array on nonhuman model systems such as Cynomolgus macaque and great apes offers the opportunity to directly compare at single nucleotide resolution the DNA methylation profiles across evolutionary trees, including within-species comparisons of geographically diverse populations. Likewise, the potential for cross-species comparisons is most exciting, given the thousands of human DNA methylation profiles that are being created using this technology.

To better benchmark the performance of the Infinium 450K array we compared resulting data directly to those obtained using RRBSseq performed on the same samples. Even with aggressive filtering we do not achieve the agreement between Infinium and RRBS that has been seen for human tissues *i.e.*, correlation of 0.97 and 84% of probes within 10% methylation error (Pan *et al.* 2012). This discrepancy could be due to experimental variation or sequencing errors in the unfinished Cynomologus genome. Invalid sequence (or indeed polymorphism) would affect the accuracy of our alignment calls and could have led to the inclusion of mismatched probes. Nevertheless, this cross-technology comparison strongly supports the technical and biological validity of the Cynomolgus macaque DNA methylation profiles obtained from the Infinium 450K array.

Various sequencing methods for profiling DNA methylation at the genome-wide or whole-genome scale are available, however, these are expensive and require high quality genome sequence as a reference. The popular platforms include RRBS and MeDIP-seq. MeDIP-seq or MBD-seq uses an antibody or methyl-binding protein to create a genomic library of methylated regions that are then sequenced. It offers whole-genome coverage, but has poor resolution since methylation is detected at regions rather than individual residues, and hybridization to the antibody or methyl-binding protein is not necessarily linear to percent methylation. Read-counts must be corrected for CpG density, which is an inexact process (Down *et al.* 2008). RRBS uses methylation-sensitive restriction enzymes to create a genomic fragment library of methylated regions that are then bisulfite converted and sequenced. RRBS has single-CpG level resolution and offers much higher coverage than Infinium (approximately 6 million CpGs) but tends to bias toward regions that are moderately or highly methylated and does not cover the hypomethylated regions that are thought to be important in inter-individual variation. Interestingly a recent analysis on genome-wide, interindividual variation in DNA methylation in humans suggests that despite the greater coverage of RRBS, RRBSseq and Infinium 450K arrays capture a comparable percentage of variably methylated CpGs (Ziller *et al.* 2013).

Array-based methods offer researchers familiar protocols, simpler data analysis and relatively low costs compared with sequencing. The Infinium array also generally requires less starting material than standard sequence based methods. Each Infinium 450K run costs about half that of RRBS and MeDIP-seq. This cost-saving would be most pertinent to large epigenome-wide association studies involving Cynomolgus macaques. Finally, researchers who wish to profile the DNA methylome in Cynomolgus macaque tissues should use the Infinium 450K array with some caution, using the filters described here and confirming locus-specific results with an independent technique. We suggest that these considerations permit the use of the Human Infinium 450K array with Cynomolgus macaque tissues, an approach that should, among other possibilities, enable affordable and powerful studies of tissue specificity in a complex, non-human primate model with considerably larger sample sizes than would be afforded by sequencing protocols.

## Supplementary Material

Supporting Information
