# Supplementary material for: Infinium Monkeys: Infinium 450K Array for the Cynomolgus macaque (Macaca fascicularis)
Source: G3 (Bethesda). 2014 May 8;4(7):1227–34. doi: 10.1534/g3.114.010967 (PMC4455772; doi:10.1534/g3.114.010967)
Supplement: Supporting Information [file supp_4_7_1227__index.html]

Infinium Monkeys: Infinium 450K Array for the Cynomolgus macaque (Macaca fascicularis) — Supporting Information 

# Infinium Monkeys: Infinium 450K Array for the Cynomolgus macaque (*Macaca fascicularis*)

## Supporting Information for Ong *et al.*, 2014

**Files in this Data Supplement:**

- Supporting Information - Figures S1-S7, Tables S1-S2, and File S1 (PDF, 901 KB)
- Figure S1 - (a) Average green and red negative control probe intensities with standard deviations for the twelve Infinium 450K data for the Cynomolgus macaque muscle tissues. (PDF, 371 KB)
- Figure S2 - (a) Scatterplot of Infinium 450K data for Cynomolgus macaque muscle sample versus technical replicate of sample. (b) Scatterplot of Infinium 450K data for two independent Cynomolgus macaque muscle samples. (PDF, 547 KB)
- Figure S3 - (a) Scatterplot of Infinium 450K data versus RRBS data for one sample stratified by Infinium type I and type II probes. (b) Percent of probes with varying percent error between RRBS and Infinium data with type I or type II probes. (PDF, 506 KB)
- Figure S4 - (a) Scatterplot of Infinium 450K data versus RRBS data for all samples stratified by polymorphic and non-polymorphic probes. (b) Percent of probes with varying percent error between RRBS and Infinium data with non-polymorphic or polymorphic probes. (PDF, 246 KB)
- Figure S5 - Scatter plot of inter-individual differences between two samples as measured by the Infinium 450K array or RRBS. (PDF, 153 KB)
- Figure S6 - Correlation of Infinium 450K and RRBS data for selected probes sequences with bitscores in the indicative ranges. (PDF, 161 KB)
- Figure S7 - Number of array probes using different bitscore thresholds. (PDF, 140 KB)
- Table S1 - Per-sample correlation between RRBS and Infinium 450K data and % probes with less than 10% methylation difference between the two technologies stratified across different alignment scores. (PDF, 154 KB)
- Table S2 - Various alignment quality metrics for Type 1 and Type 2 Infinium probes. (PDF, 122 KB)
- File S1 - Infinium 450K annotation file for Cynomolgus macaque. (.xlsx, 41 MB)
