# Supplementary material for: Infinium Monkeys: Infinium 450K Array for the Cynomolgus macaque (Macaca fascicularis)
Source: G3 (Bethesda). 2014 May 8;4(7):1227–34. doi: 10.1534/g3.114.010967 (PMC4455772; doi:10.1534/g3.114.010967)
Supplement: Supporting Information [file supp_g3.114.010967_010967SI.pdf]

G3: Genes | Genomes | Genetics

Infinium Monkeys: Infinium 450K array for the Cynomolgus macaque (*Macaca fascicularis*)

Mei-Lyn Ong[*], Peck Yean Tan[*], Julia L MacIsaac[§], Sarah M Mah[§], Jan Paul Buschdorf*, Clara Y Cheong*, Walter Stunkel*, Louiza Chan*, Peter D Gluckman*, Keefe Chng*, Michael S. Kobor[§], Michael J Meaney*[,†], Joanna D Holbrook*
[*] Singapore Institute of Clinical Sciences (SICS), A*STAR, Singapore
[§]Centre for Molecular Medicine and Therapeutics, Child and Family Research Institute, University of British Columbia, Canada
[†]Ludmer Centre for Neuroinformatics and Mental Health, Douglas Institute, McGill University, Montreal, Canada

GSE IDs: GSE52944 (Infinium 450K data), GSE53596 (RRBS data)

Corresponding Author:
Joanna Holbrook
Singapore Institute for Clinical Sciences (SICS), Brenner Centre for Molecular Medicine, 30 Medical Drive, Singapore 117609
+65 6407 0688
Joanna_Holbrook@sics.a-star.edu.sg

DOI: 10.1534/g3.114.010967

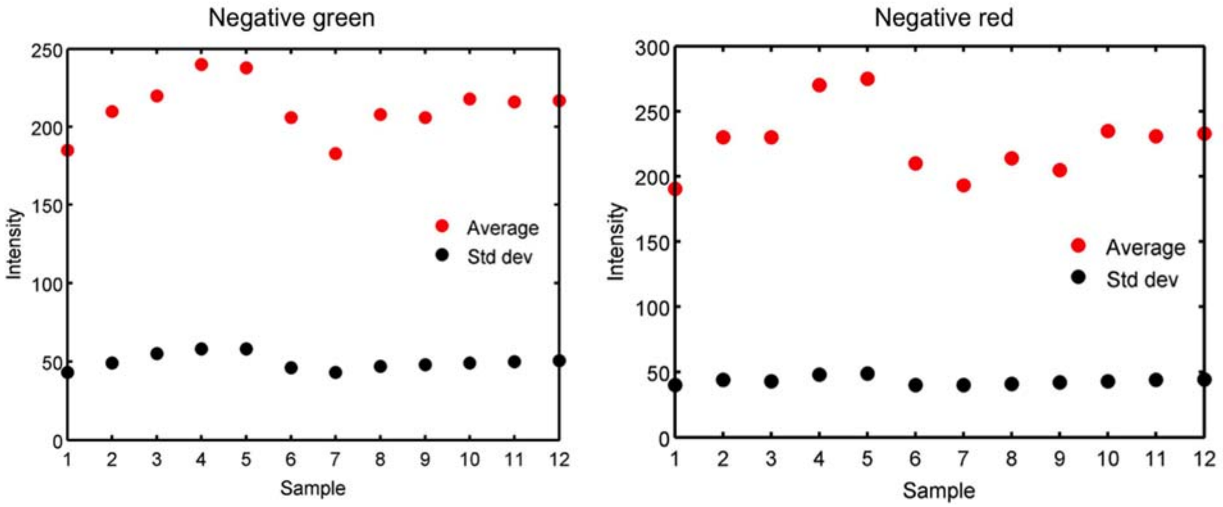

Figure S1 (a) Average green and red negative control probe intensities with standard deviations for the twelve Infinium 450K data for the Cynomolgus macaque muscle tissues.

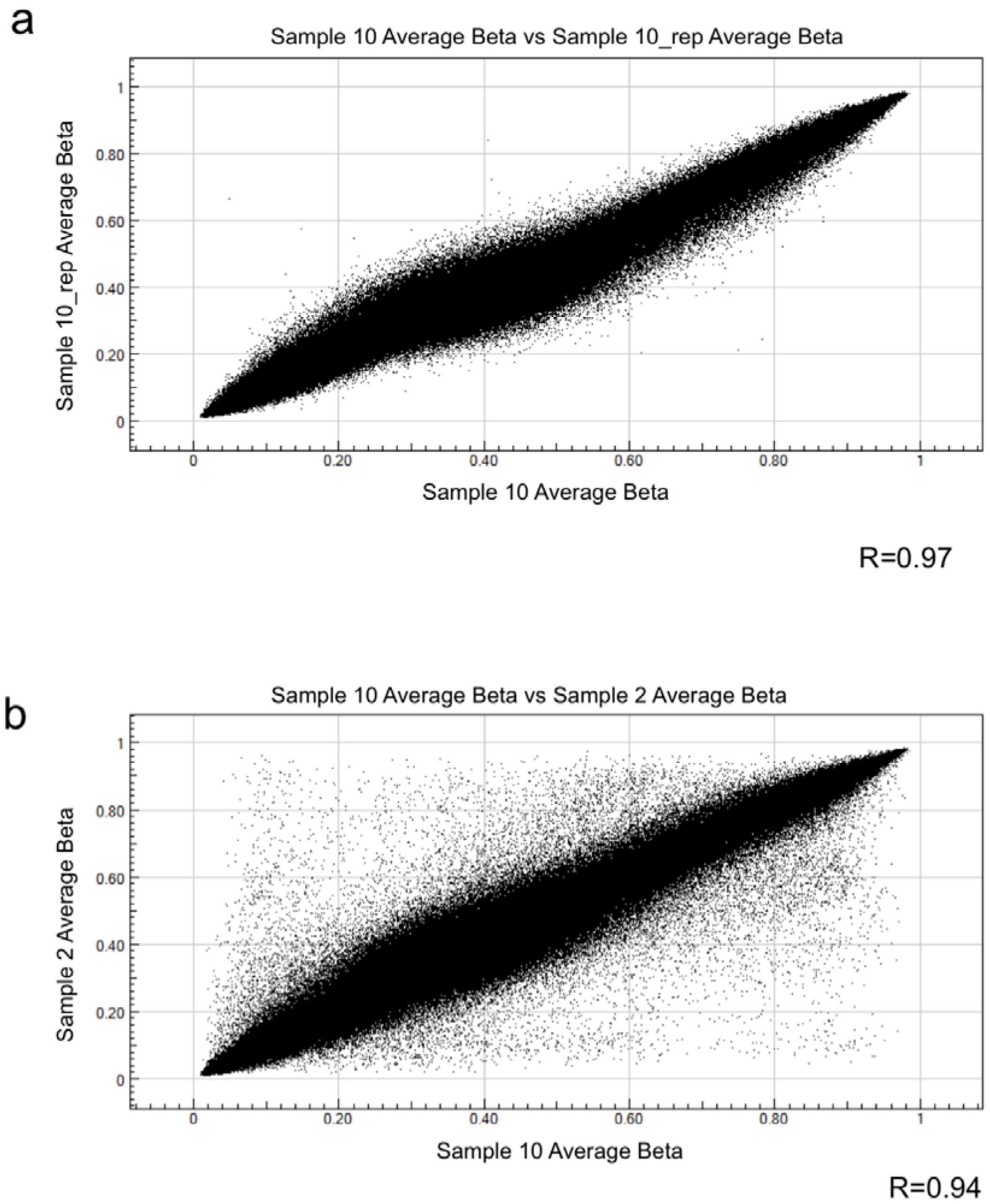

Figure S2 (a) Scatterplot of Infinium 450K data for Cynomolgus macaque muscle sample versus technical replicate of sample. (b) Scatterplot of Infinium 450K data for two independent Cynomolgus macaque muscle samples.

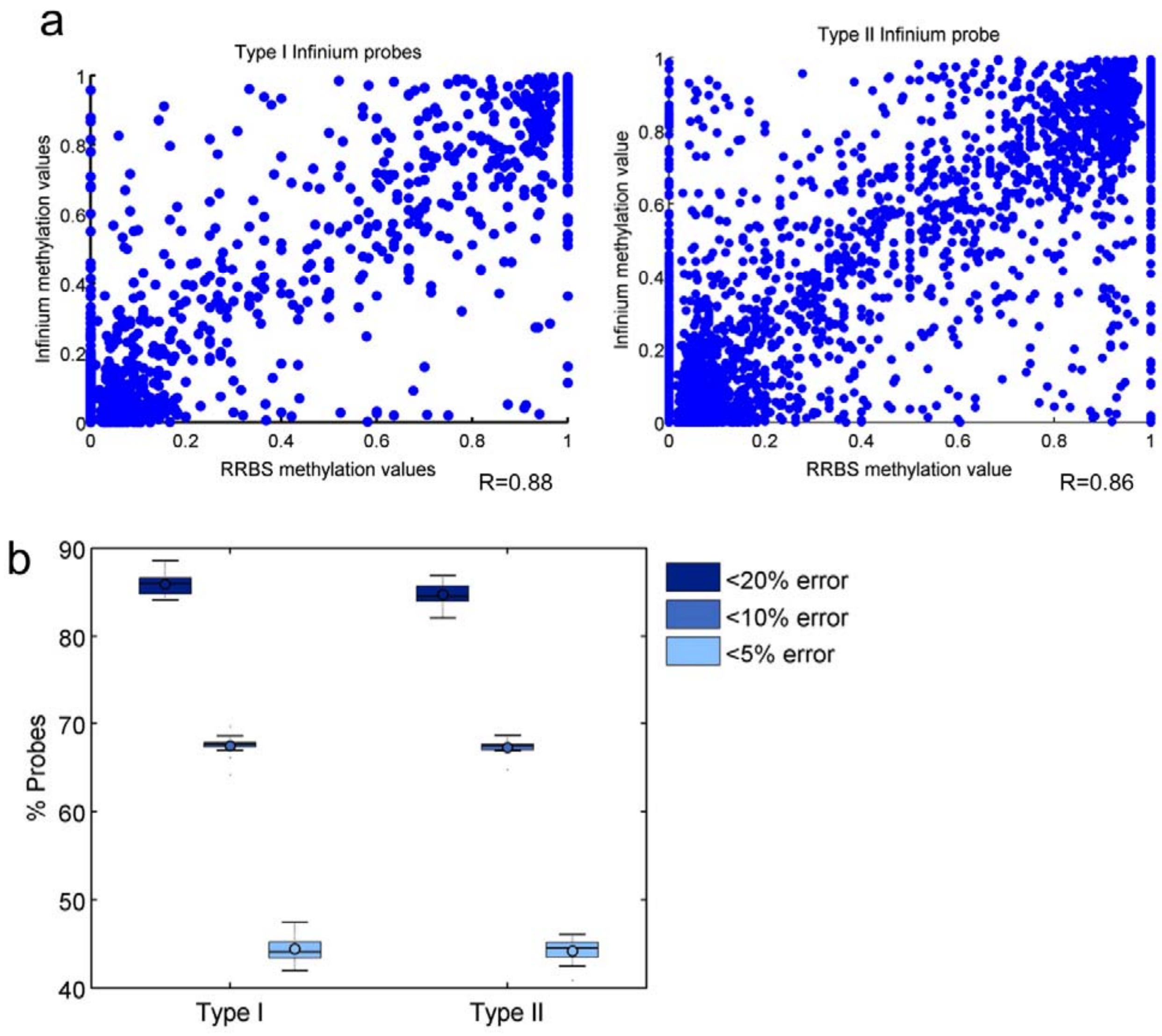

Figure S3 (a) Scatterplot of Infinium 450K data versus RRBS data for one sample stratified by Infinium type I and type II probes. (b) Percent of probes with varying percent error between RRBS and Infinium data with type I or type II probes.

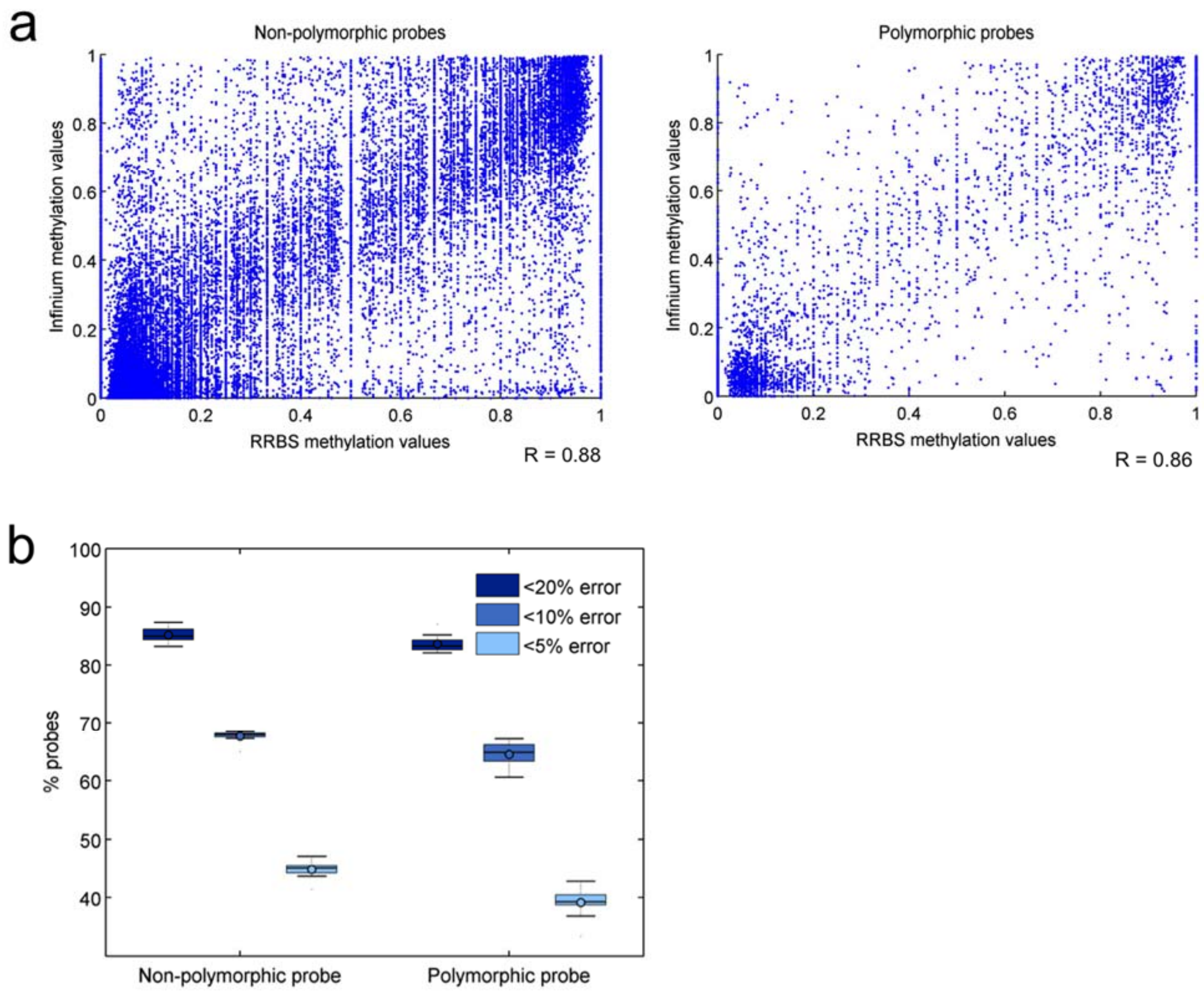

Figure S4 (a) Scatterplot of Infinium 450K data versus RRBS data for all samples stratified by polymorphic and non-polymorphic probes. (b) Percent of probes with varying percent error between RRBS and Infinium data with non-polymorphic or polymorphic probes.

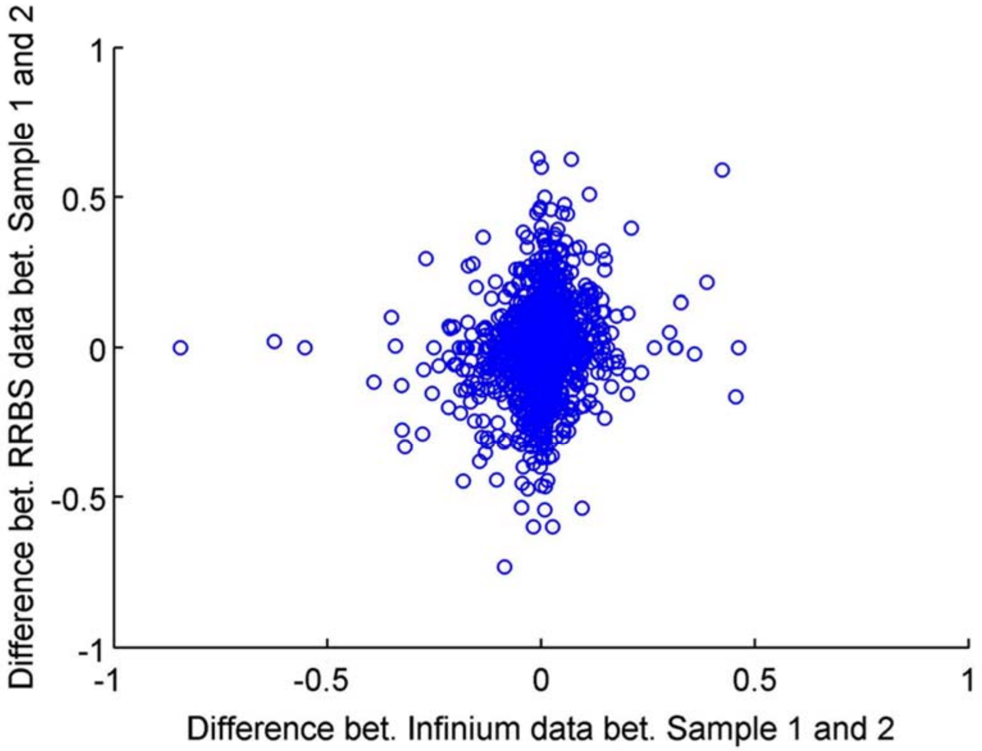

Figure S5 Scatter plot of inter-individual differences between two samples as measured by the Infinium 450K array or RRBS.

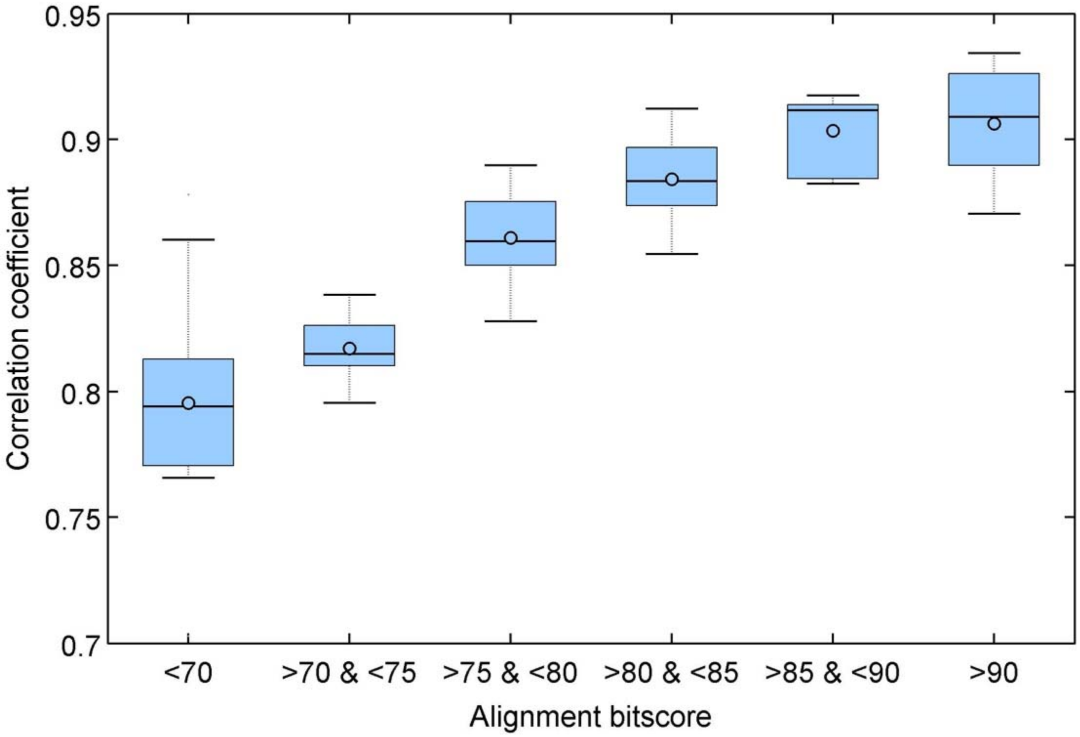

Figure S6 Correlation of Infinium 450K and RRBS data for selected probes sequences with bitscores in the indicative ranges.

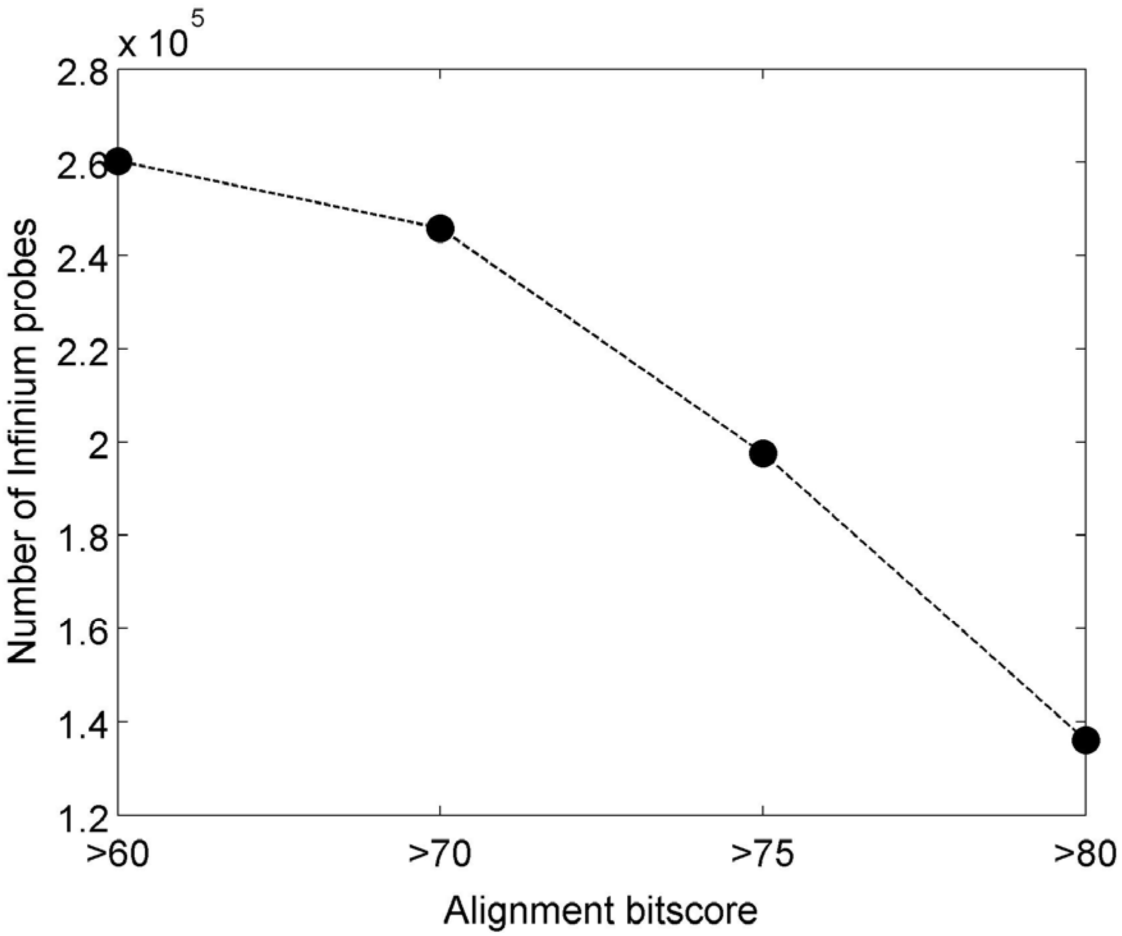

Figure S7 Number of array probes using different bitscore thresholds.

Table S1 Per-sample correlation between RRBS and Infinium 450K data and % probes with less than 10% methylation difference between the two technologies stratified across different alignment scores.

Alignment score	Sample number	1	2	3	4	5	6	7	8	9	10	10_rep	12
Bit score < 70	Correlation	0.70	0.88	0.78	0.79	0.86	0.81	0.77	0.81	0.80	0.77	0.77	0.80
	% Probes <10% error	54.7	63.6	55.7	64.0	62.1	64.5	57.8	62.5	64.3	59.2	60.0	63.9
Bit score > 70	Correlation	0.86	0.88	0.86	0.88	0.87	0.90	0.85	0.88	0.90	0.88	0.88	0.90
	% Probes <10% error	68.8	69.0	65.9	69.1	69.1	68.8	68.7	69.2	68.2	69.0	69.1	68.4
Bit score > 80	Correlation	0.88	0.90	0.88	0.91	0.88	0.92	0.88	0.90	0.92	0.90	0.90	0.92
	% Probes <10% error	70.4	69.7	66.9	70.6	70.8	70.2	71.4	70.3	69.8	70.3	71.8	69.6

Table S2 Various alignment quality metrics for Type 1 and Type 2 Infinium probes.

Alignment quality	Infinium probe	
	Type 1	Type 2
Average % identity	96.1	96.3
Average bitscore	81.7	81.8
Average E-value	$3.4e^{-12}$	$2.6e^{-12}$

File S1

Infinium 450K annotation file for Cynomolgus macaque.

Available for download as an Excel file at http://www.g3journal.org/lookup/suppl/doi:10.1534/g3.114.010967/-/DC1.
