# Supplementary material for: Infinium Monkeys: Infinium 450K Array for the Cynomolgus macaque (Macaca fascicularis)
Source: G3 (Bethesda). 2014 May 8;4(7):1227–34. doi: 10.1534/g3.114.010967 (PMC4455772; doi:10.1534/g3.114.010967)
Supplement: Supporting Information [file supp_g3.114.010967_FigureS1.pdf]

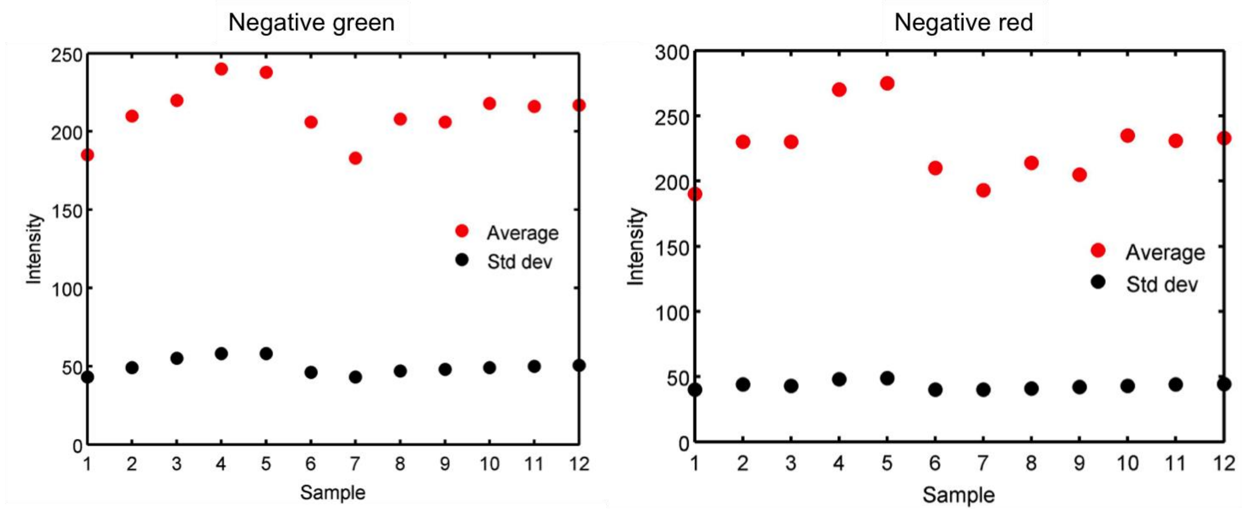

Figure S1 (a) Average green and red negative control probe intensities with standard deviations for the twelve Infinium 450K data for the Cynomolgus macaque muscle tissues.
