# Supplementary material for: Infinium Monkeys: Infinium 450K Array for the Cynomolgus macaque (Macaca fascicularis)
Source: G3 (Bethesda). 2014 May 8;4(7):1227–34. doi: 10.1534/g3.114.010967 (PMC4455772; doi:10.1534/g3.114.010967)
Supplement: Supporting Information [file supp_g3.114.010967_FigureS2.pdf]

a

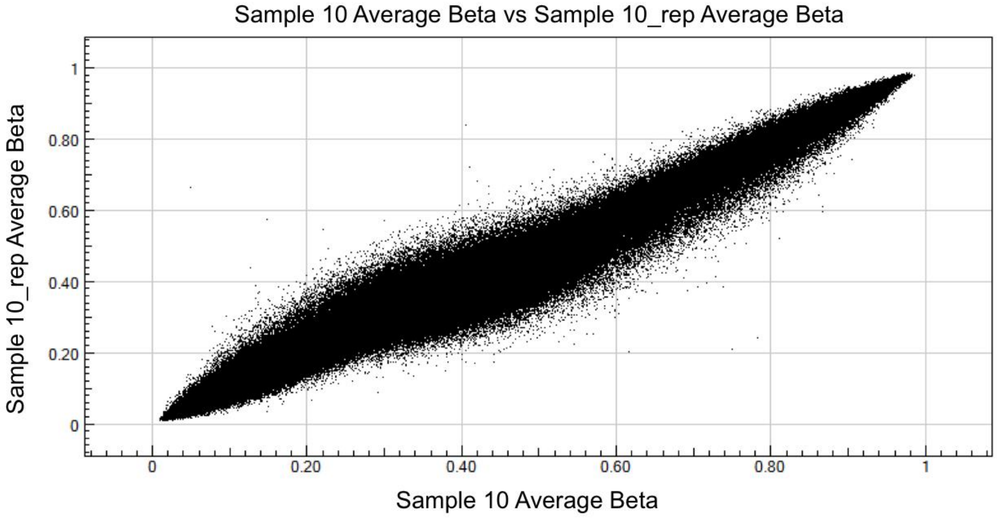

R=0.97

b

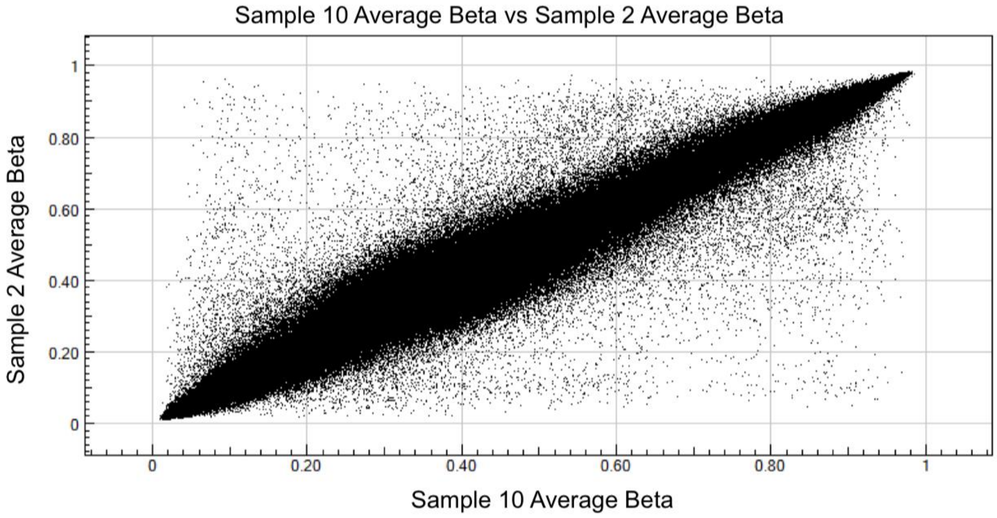

R=0.94

Figure S2 (a) Scatterplot of Infinium 450K data for Cynomolgus macaque muscle sample versus technical replicate of sample. (b) Scatterplot of Infinium 450K data for two independent Cynomolgus macaque muscle samples.
