# Supplementary material for: Infinium Monkeys: Infinium 450K Array for the Cynomolgus macaque (Macaca fascicularis)
Source: G3 (Bethesda). 2014 May 8;4(7):1227–34. doi: 10.1534/g3.114.010967 (PMC4455772; doi:10.1534/g3.114.010967)
Supplement: Supporting Information [file supp_g3.114.010967_FigureS4.pdf]

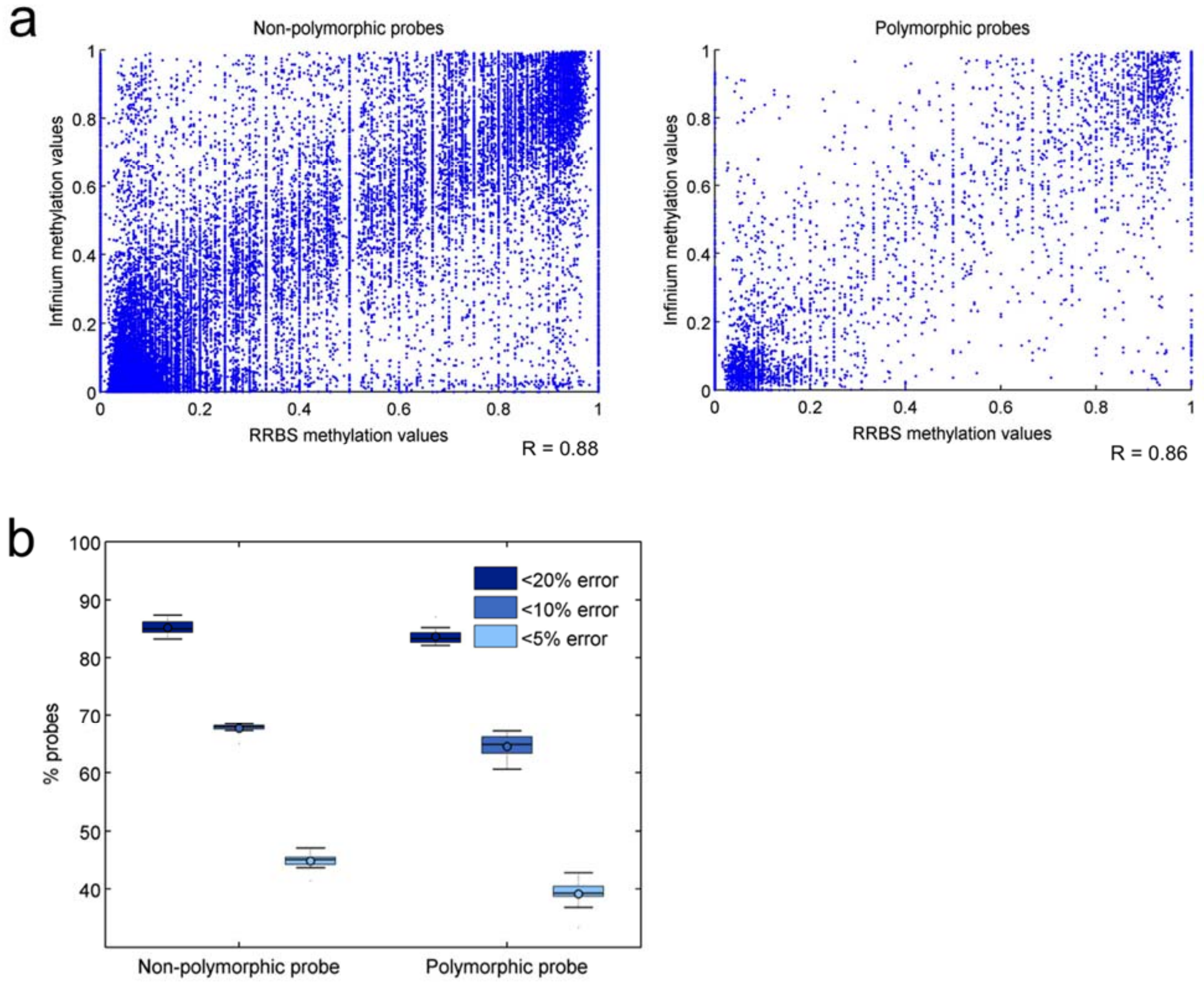

Figure S4 (a) Scatterplot of Infinium 450K data versus RRBS data for all samples stratified by polymorphic and non-polymorphic probes. (b) Percent of probes with varying percent error between RRBS and Infinium data with non-polymorphic or polymorphic probes.
