# Supplementary figures and images for: Infinium Monkeys: Infinium 450K Array for the Cynomolgus macaque (Macaca fascicularis)
Source: G3 (Bethesda). 2014 May 8;4(7):1227–34. doi: 10.1534/g3.114.010967 (PMC4455772; doi:10.1534/g3.114.010967)

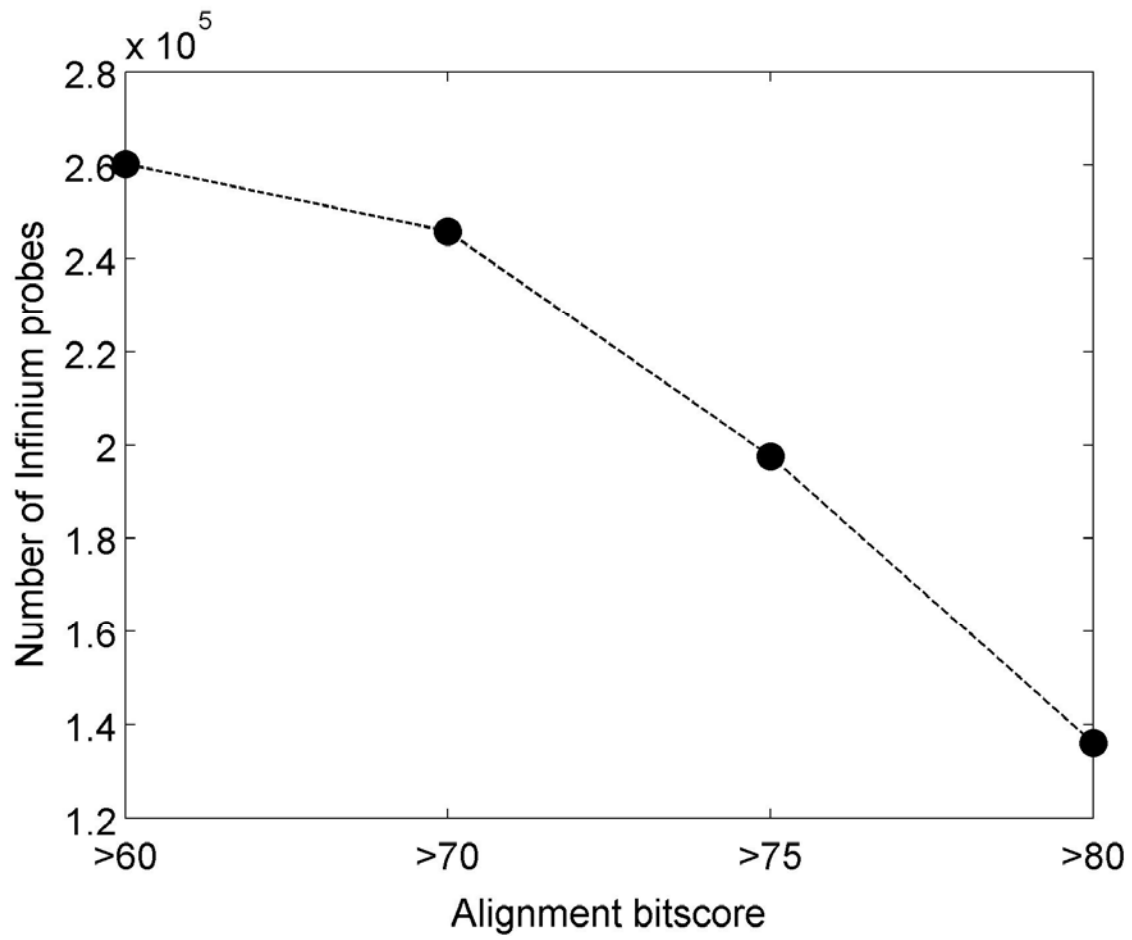

**Figure S7** Number of array probes using different bitscore thresholds.

Supplement: Supporting Information [file supp_g3.114.010967_FigureS7.pdf]
