# Supplementary material for: Infinium Monkeys: Infinium 450K Array for the Cynomolgus macaque (Macaca fascicularis)
Source: G3 (Bethesda). 2014 May 8;4(7):1227–34. doi: 10.1534/g3.114.010967 (PMC4455772; doi:10.1534/g3.114.010967)
Supplement: Supporting Information [file supp_g3.114.010967_TableS1.pdf]

Table S1 Per-sample correlation between RRBS and Infinium 450K data and % probes with less than 10% methylation difference between the two technologies stratified across different alignment scores.

Alignment score	Sample number	1	2	3	4	5	6	7	8	9	10	10_rep	12
Bit score < 70	Correlation	0.70	0.88	0.78	0.79	0.86	0.81	0.77	0.81	0.80	0.77	0.77	0.80
	% Probes <10% error	54.7	63.6	55.7	64.0	62.1	64.5	57.8	62.5	64.3	59.2	60.0	63.9
Bit score > 70	Correlation	0.86	0.88	0.86	0.88	0.87	0.90	0.85	0.88	0.90	0.88	0.88	0.90
	% Probes <10% error	68.8	69.0	65.9	69.1	69.1	68.8	68.7	69.2	68.2	69.0	69.1	68.4
Bit score > 80	Correlation	0.88	0.90	0.88	0.91	0.88	0.92	0.88	0.90	0.92	0.90	0.90	0.92
	% Probes <10% error	70.4	69.7	66.9	70.6	70.8	70.2	71.4	70.3	69.8	70.3	71.8	69.6
