# Supplementary material for: Infinium Monkeys: Infinium 450K Array for the Cynomolgus macaque (Macaca fascicularis)
Source: G3 (Bethesda). 2014 May 8;4(7):1227–34. doi: 10.1534/g3.114.010967 (PMC4455772; doi:10.1534/g3.114.010967)
Supplement: Supporting Information [file supp_g3.114.010967_TableS2.pdf]

Table S2 Various alignment quality metrics for Type 1 and Type 2 Infinium probes.

Alignment quality	Infinium probe	
	Type 1	Type 2
Average % identity	96.1	96.3
Average bitscore	81.7	81.8
Average E-value	$3.4e^{-12}$	$2.6e^{-12}$
